# Physiochemical properties of short‐term frying oil for chicken wing and its oxidative stability in an oil‐in‐water emulsion

**DOI:** 10.1002/fsn3.1355

**Published:** 2019-12-17

**Authors:** He Jiang, Wenwei Chen, Zhenbao Jia, Fei Tao

**Affiliations:** ^1^ Center for Food Safety & Quality Hangzhou Institute for Food and Drug Control Hangzhou China; ^2^ Key Laboratory of Marine Food Quality and Hazard Controlling Technology of Zhejiang Province China Jiliang University Hangzhou China; ^3^ College of Standardization China Jiliang University Hangzhou China

**Keywords:** deterioration, frying oil, oxidative stability, physiochemical property

## Abstract

In this study, the physiochemical properties of corn oil and its oxidative stability in an O/W emulsion were studied following short‐term (120 min) deep‐frying of chicken wing. The results showed that the levels of polyunsaturated fatty acids in corn oil decreased after frying. Furthermore, total polar compound content in frying oil was significantly increased to 11.3%. Fourier transform infrared spectra (FTIR) indicated that hydrolysis and oxidation reactions involving triglycerides occurred after frying. Additionally, the increased a* and b* values demonstrated that deep‐frying greatly enhanced the intensity of the red and yellow colors of corn oil. Frying reduced the oxidative stability of corn oil in an O/W emulsion as determined by the peroxide value and acid value. These findings indicated that short‐term deep‐frying of chicken wing deteriorated the quality of corn oil and decreased its oxidative stability in an O/W emulsion. Consumers should consider the potential hazards of food containing short‐term deep‐frying oil.

## Graphical abstract

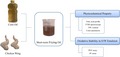

## 1 INTRODUCTION

Deep‐frying is a popular cooking method worldwide, in which food is submerged in hot oil at temperatures ≥180°C. Because oxygen and water are present in food, triglycerides at high cooking temperatures undergo numerous chemical reactions including polymerization, oxidation, isomerization, and hydrolysis (Gertz, Aladedunye, & Matthäus, 2014; Kmiecik, Kobus‐Cisowska, & Korczak, 2017; Mekawi, Sharoba, & Ramadan, 2019). Thus, deep‐frying, either continuously or in batches, may cause oil degradation and the accumulation of some toxic molecules. Deep‐frying alters the chemical composition of oil, thus affecting its quality (Li, Li, Wang, Cao, & Liu, 2017; Li, Yu, et al., 2017; Ramadan, 2015). From an economic perspective, frying oil in domestic cooking processes is reused to prepare other foods. Therefore, changes in the physiochemical properties of short‐term deep‐frying oil have important effects on food quality.

Lipid oxidation is an undesirable chemical reaction occurring in foods containing oil, as it has a series of adverse effects on the aroma, flavor, and nutritional value of food products (Shahidi & Zhong, 2010). Oil‐in‐water (O/W) emulsions are the basis of many common foods, such as salad dressings, hollandaise, and mayonnaise. O/W emulsions are composed of an aqueous phase and dispersed lipid droplets covered with amphiphilic emulsifier (Kontogiorgos, 2017). Lipids in O/W emulsions are highly susceptible to oxidation because of their higher interfacial area, which may enhance the interaction between lipid droplets and pro‐oxidants in the aqueous phase (Lomova, Sukhorukov, & Antipina, 2010). Oxidative stability has long been recognized as an essential requirement for food O/W emulsions. The oxidative stability of deep‐frying oils in bulk oil systems during storage has been well documented (Aladedunye & Matthäus, 2014; Karakaya & Şimşek, 2011). However, little information is available for the oxidative stability of deep‐frying oil in O/W emulsions.

In the past decade, numerous studies have explored the chemical reactions occurring during the deep‐frying process; however, these reactions are not completely understood. Several authors proposed that the types of food subjected to frying greatly influenced the properties of frying oil (Bansal, Zhou, Barlow, Lo, & Neo, 2010; Orozco‐Solano, Priego‐Capote, & Luque de Castro, 2011). Chicken wing is a very common and generally acceptable food in the diet. Deep‐frying often makes chicken wing more palatable by conferring distinctive sensory qualities, including crisp texture, golden color, and good flavor. However, few studies have examined the relationship between the physicochemical properties of frying oil of chicken wing and its oxidative stability in an O/W emulsion.

Thus, this study was conducted to evaluate the physiochemical properties of short‐term deep‐frying oil in which chicken wing had been prepared. The oxidative stability of frying oil in a whey protein‐stabilized O/W emulsion was also evaluated.

## 2 MATERIALS AND METHODS

### 2.1 Chemicals and reagents

Refined corn oil and chicken wing were purchased from a local market. Whey protein isolate was obtained from Hilmar Cheese Co. Silica gel powder (100–200 mesh) was provided by Qingdao Haiyang Chemical Co., Ltd. All chemical solvents used in this study were analytical grade and purchased from Mike Chemical Co., Ltd.

### 2.2 Preparation of short‐term deep‐frying oil

Corn oil (6 L) in a Dongbei EF‐101V deep‐fryer was heated to 180°C, and then 0.5 kg of whole chicken wing was added and fried for 20 min. After each frying operation, the oil was cooled to lower than 60°C. Six batches of chicken wing were subjected to thermo‐frying in the same oil. Thus, the total frying period was 120 min. The frying oil was cooled to room temperature and then stored at −20°C until analysis. Unheated corn oil (fresh oil) was used as a control sample.

### 2.3 Measurement fatty acid profile

Fatty acid methyl esters (FAMEs) were formed by the esterization method as described by Kim et al. (2015). The FAMEs were analyzed using an Agilent Technologies 7890N gas chromatograph equipped with a flame ionization detector. The gas chromatography column was an Agilent DB‐23 capillary column (60 m × 0.25 mm i.d. × 0.2 mm film thickness). The initial column temperature was 165°C for 25 min and was then increased to 195°C at a gradient of 5°C/min. Helium (1 ml/min) was used as the carrier gas, and the injected sample volume was 1 μl. The temperatures of the detector and injector were maintained at 250°C. FAMEs were identified by comparing their retention times with those of FAME standards, and the relative contents were calculated as w/w%.

### 2.4 Fourier transform infrared spectra spectroscopy

Fourier transform infrared spectroscopy (FTIR) was performed using a Shimadzu IRAffinity‐1 FTIR spectrometer. The oil samples were applied to the surface of KBr pellets. After 24 hr of vacuum drying, the FTIR spectra of oil samples were recorded in the region of 4,000–1,660 cm^−1^, with 120 scans acquired at a resolution of 4 cm^−1^.

### 2.5 Measurement of total polar compound content

The total polar compound (TPC) contents in the oil samples were determined by gravimetric measurement by silica gel column chromatography as described by Juárez, Osawa, Acuña, Sammán, and Gonçalves (2011). Nonpolar fractions in the samples were separated by silica column chromatography using petroleum ether/diethyl ether (87:13, v/v).

### 2.6 Color measurement

Color was determined using a Minolta CR‐300 colorimeter. The oil samples were transferred to a sample dish and positioned in the light path to monitor the color parameter values of L*, a*, and b*.

### 2.7 Emulsion preparation

Whey protein isolate (10 g) was suspended in 100 ml of sodium phosphate buffer (0.2 M, pH 6.0) containing sodium azide (0.1 mg/ml) and then mixed with 30 ml of oil sample. The mixture was homogenized with a GEA NS1001L2K high‐pressure homogenizer for two passes at 25 MPa.

### 2.8 Oxidative stability determination

The emulsion (0.6 g) was added to 3.0 ml of isooctane/2‐propanol (3:1, v/v) and vortexed three times for 10 s each time. The organic phase was obtained by centrifugation at 1,500 *g* for 5 min. The organic phase was dried under a stream of N_2_. The peroxide value (milliequivalents/kg oil) and acid value (KOH/g oil) of the oil samples were determined according to the AOAC Official Method (AOAC, 2010).

### 2.9 Statistical analysis

The data are expressed as the mean ± standard deviation. Differences between means were determined by one‐way analysis of variance, and the means were compared by Duncan's multiple range test. The statistical significance was set to *p* < .05. Analysis was conducted using SPSS statistical software version 13.0 (SPSS, Inc.).

## 3 RESULTS AND DISCUSSION

### 3.1 Fatty acid profile of frying oil

Frying oil, used continuously or in batches at high temperature, undergoes numerous degradation reactions. The fatty acid profile can be measured to evaluate thermo‐oxidative degradation of edible oil during deep‐frying (Hammouda, Zribi, Mansour, Matthäus, & Bouaziz, 2017; Ramadan, Afify Amer, Sulieman, & El‐Rahman, 2006). The fatty acid profiles of fresh and deep‐frying oils are shown in Table 1. Of the total fatty acids in fresh oil, 62.1% were polyunsaturated fatty acids (PUFA), among which the predominant fatty acid was linoleic acid (C_18:2n6_). A total of 27.0% of total fatty acids were monounsaturated fatty acids (MUFA), with oleic acid (C_18:1n9_) as the most abundant fatty acid. The level of saturated fatty acids (SFA) in fresh oil was 10.9%, consisting mainly of palmitic acid (C_16:0_). After 120 min of deep‐frying, the level of PUFA decreased, whereas those of SFA and MUFA increased. A similar trend was observed by Li, Li, et al. (2017), Li, Yu, et al. (2017). The decreased level of PUFA in deep‐frying oil can be explained by the sensitivity of oil to thermal oxidation, which resulted in the generation of conjugated dienes and trienes during frying (Abdulkarim, Long, Lai, Muhammad, & Ghazali, 2007). PUFA is known to play a crucial role in human nutrition by preventing health disorders. Thus, from a nutritional perspective, short‐term deep‐frying of chicken wing decreased the nutritional quality of the oil. Additionally, palmitic acid level was increased after frying, which has been reported by Bansal et al. (2010), who revealed that oxidative deterioration of linoleic acid under high‐temperature condition led to the formation of palmitic acid. Hence, the increase in palmitic acid level in frying oil can be ascribed to the reduction in linoleic acid level. Because palmitic acid is more stable against oxidation, whereas linoleic acid is more susceptible to oxidation, the C_18:2n6_/C_16:0_ ratio may be an effective indicator of the degree of edible oil deterioration (Hammouda, Triki, Matthäus, & Bouaziz, 2018; Zribi, Jabeur, Matthäus, & Bouaziz, 2016). As shown in Table 1, the C_18:2n6_/C_16:0_ ratio was decreased from 9.64 for fresh oil to 7.09 for frying oil, indicating that oil deterioration occurred during short‐term deep‐frying.

**Table 1:** Fatty acid profile (%) of fresh and deep‐frying oils

	Fresh oil	Frying oil
C_8:0_	ND	0.11
C_14:0_	0.07	0.13
C_15:0_	0.02	ND
C_16:0_	6.39	7.97
C_16:1_	0.11	0.29
C_17:0_	0.03	0.04
C_18:0_	3.33	3.85
C_18:1n9_	26.83	29.10
C_18:2n6_	61.58	56.51
C_18:3n3_	0.09	0.09
C_20:0_	0.25	0.36
C_20:1_	0.15	0.19
C_20:2_	0.05	0.07
C_20:5n3_	0.26	0.28
C_22:0_	0.73	0.87
C_22:2n6_	0.02	0.04
C_23:0_	0.03	0.04
C_24:1_	0.06	0.06
SFA	10.9	13.4
MUFA	27.0	29.6
PUFA	62.1	57.0
C_18:2n6_/C_16:0_	9.64	7.09

Abbreviation: ND, not detected (below detection limit).

### 3.2 Fourier transform infrared spectra spectra of frying oil

**Figure 1:**
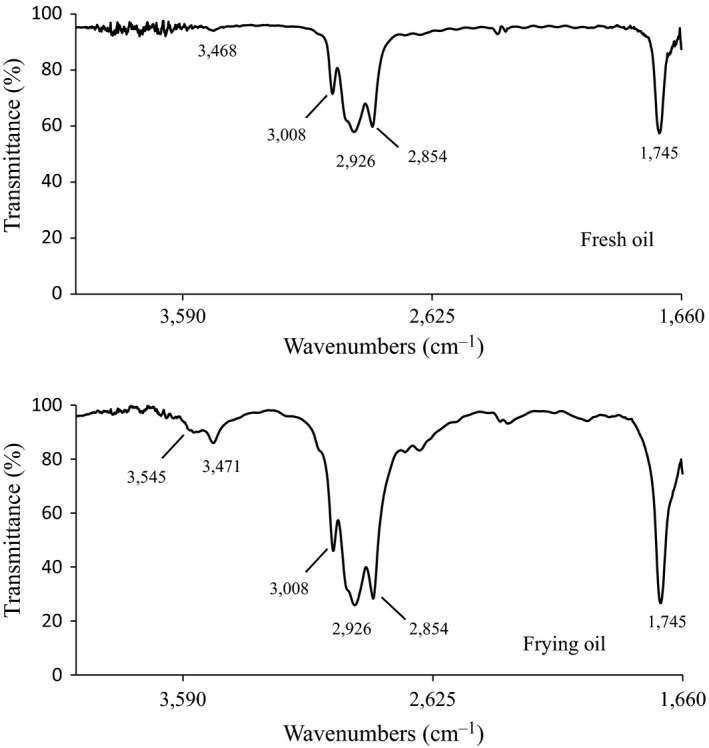
Fourier transform infrared spectra (FTIR) spectra of fresh and frying oils

Fourier transform infrared spectra spectroscopy provides detailed insight into the changes in chemical groups representative of chemical reactions that occur during deep‐frying, particularly hydrolysis and oxidation reactions. Thus, FTIR spectroscopy is useful for exploring the degradation of frying oils (Chen et al., 2018; Innawong, Mallikarjunan, Irudayaraj, & Marcy, 2004). The FTIR spectra of oil samples are shown in Figure 1. The spectrum of fresh oil showed four strong absorption bands, assigned to *cis* carbon–carbon double bonds stretching (3,008 cm^−1^), asymmetric –CH_2_ stretching (2,926 cm^−1^), symmetric –CH_2_ stretching (2,854 cm^−1^), and ester carbonyl stretching (1,745 cm^−1^). The small band at 3,468 cm^−1^ was assigned to the overtone of the glyceride ester carbonyl group. The band intensity of the glyceride ester carbonyl group was greatly enhanced as compared to that for fresh oil because of hydrolysis of the ester linkage and generation of free fatty acids (Tena, Aparicio‐Ruiz, & García‐González, 2013). A new band at 3,545 cm^−1^, assigned to hydroxy groups, was observed in the spectrum of deep‐frying oil. It is well‐known that the oxidation of oil causes degradation of hydroperoxides, leading to the formation of secondary oxidation products, some of which contain hydroxy groups (Guillén & Cabo, 1999). Thus, the appearance of a hydroxy absorption band in deep‐frying oil reflected the formation of hydroperoxides produced in an oxidation reaction. The change in the FTIR spectrum indicated that the hydrolysis and oxidation reactions of triglycerides occurred during short‐term deep‐frying.

### 3.3 Total polar compound content of frying oil

**Figure 2:**
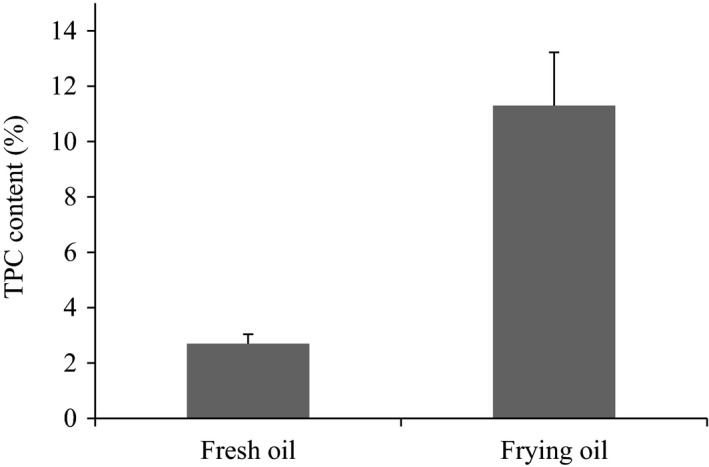
Total polar compound (TPC) contents of fresh and frying oils. Results are expressed as the mean ± standard deviation; *n* = 3

During deep‐frying, oil is subjected to high temperatures, thus producing numerous oxidation products. Because oxidation reactions enhance the polarity of lipid molecules, the TPC content is a reliable parameter for evaluating the quality of frying oil (Farhoosh & Tavassoli‐Kafrani, 2010; Hassanien & Sharoba, 2014). This method provides the most accurate examination of the thermo‐oxidative degradation degree of triglyceride during the oil frying process and offers direct information on the total amount of newly formed reaction compounds with higher polarity than triglyceride (Zribi et al., 2016). Most countries in the European Union have established a maximum TPC level of 25%. Figure 2 shows the change in TPC content after deep‐frying. The TPC content of fresh oil was only 2.7%, which is consistent with the results of Warner and Knowlton (1997). The content of TPC in short‐term deep‐frying oil reached 11.3%, which is significantly higher than that in fresh oil (*p* < .05). Although, in the present study, the TPC content of frying oil did not exceed the maximum limit for most European Union countries, the significantly increased TPC level indicated that thermo‐oxidative reactions occurred at the frying temperature, leading to the generation of polar compounds such as free fatty acids, triglyceride monomer, dimer, and polymer (Houhoula, Oreopoulou, & Tzia, 2003).

### 3.4 Color of frying oil

Another indicator used to evaluate frying oil quality is the color index, which is closely associated with consumer acceptance. In this study, the color changes in deep‐frying oil were analyzed by measuring the L* (brightness/darkness), a* (redness/greenness), and b* (yellowness/blueness) values. The color parameters of oil samples are shown in Table 2. The L* value slightly decreased from 26.94 to 25.82 (*p* > .05), indicating that the darkness of frying oil was comparable to that of fresh oil. Several previous studies showed that Maillard and caramelization reactions at high temperature are associated with the darkening of frying oil (Maskan, 2003; Ngadi, Li, & Oluka, 2007). Thus, the stability of darkness can be attributed to the low rates of these reactions. In contrast, the a* and b* values were significantly increased to −1.51 and 7.91 after deep‐frying (*p* < .05), respectively, demonstrating that frying oil had a higher intensity of red and yellow colors compared to fresh oil. This result is in accordance with previous findings, in which frying of chicken drumsticks considerably enhanced the intensity of the red and yellow color of oil (Udomkun, Innawong, Siasakul, & Okafor, 2018). These color alterations can be explained by the occurrence of oxidation, polymerization, and pyrolysis reactions (Lin, Akoh, & Reynolds, 2001); the reaction products contributed to changing the color of the oil.

**Table 2:** Color parameters of fresh and deep‐frying oils

	Fresh oil	Frying oil
L*	26.94 ± 3.14	25.82 ± 2.78
a*	−0.25 ± 0.03	−1.51 ± 0.10^*^
b*	0.63 ± 0.15	7.91 ± 1.06^*^

Note

Results are expressed as the mean ± standard deviation; *n* = 3. Significance (*p* < .05) in the same line is indicated by symbol (*).

### 3.5 Oxidative stability of frying oil in O/W emulsions

**Figure 3:**
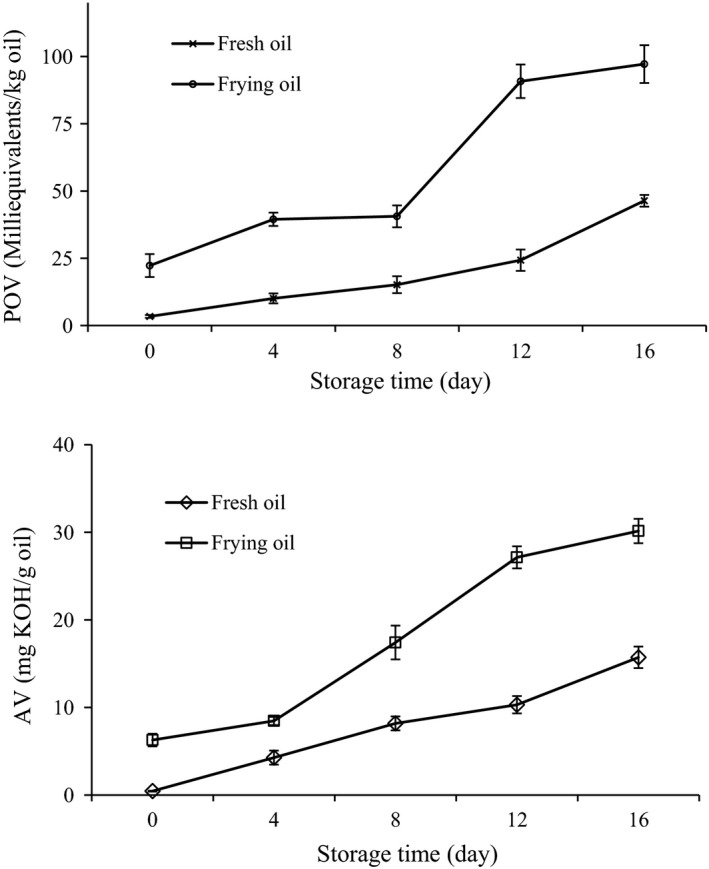
Peroxide value (POV) and acid value (AV) of fresh and frying oils in O/W emulsions during 16 days of storage. Results are expressed as the mean ± standard deviation; *n* = 3

The oxidative stability of deep‐frying oil in O/W emulsions was assessed by monitoring changes in conventional parameters of edible oil oxidation. The peroxide value (POV) indicates the levels of hydroperoxides formed from fatty acid radicals by oxidation processes. The acid value (AV) reflects the level of free fatty acids in the oil sample. The results are presented in Figure 3. The original POV of frying oil was significantly higher than that of fresh oil (*p* < .05). This observation agrees with the result of Multari, Marsol‐Vall, Heponiemi, Suomela, and Yang (2019), who reported that edible oil was prone to thermal oxidation during the frying process. The POV of frying oil clearly and rapidly increased during storage. After 16 days of storage, the POV for frying oil reached a value of 97.2, which was significantly higher than that of fresh oil (*p* < .05). The POV for fresh oil also gradually increased, but at a slower rate. Similarly, the AV of frying oil gradually increased during storage. Compared to fresh oil, the increasing in AV in frying oil was much higher. Considering the two parameters simultaneously, our findings suggest that short‐term deep‐frying deteriorated the oxidative stability of oil in the O/W emulsion. Many studies have associated the development of oil oxidation with the levels of PUFAs, which are the main targets of thermal oxidative reactions (Aladedunye & Przybylski, 2014; Nosratpour, Farhoosh, & Sharif, 2017). However, this hypothesis is inconsistent with our findings, in which deep‐frying oil, containing a lower level of PUFAs (Table 1), showed higher POV and AV parameters. In addition to being rich in PUFAs, corn oil is an important source of tocopherol (Huang, Frankel, & German, 1995). Tocopherol strongly quenches not only reactive oxygen species but also reactive nitrogen species (Forman, Davis, & Ursini, 2014; Kamal‐Eldin & Appelqvist, 1996). Therefore, this oil‐soluble vitamin effectively disrupted radical chains in lipid oxidation and played a crucial role in retarding oxidative deterioration during oil storage. It has been shown that the content of tocopherol in corn oil gradually decreases over the frying time (Simonne & Eitenmiller, 1998). Additionally, Tena, García‐González, and Aparicio (2009) found that tocopherol in edible oil was completely depleted after long‐term frying. Hence, the lower oxidative stability of frying oil may be related to the loss of tocopherol.

## 4 CONCLUSIONS

In summary, our results indicate that short‐term deep‐frying of chicken wing decreased the quality of corn oil. Corn oil was susceptible to oxidative degradation during deep‐frying, as evidenced by the decreased PUFA level, elevated TPC content, and color changes. The results obtained by FTIR demonstrated that free fatty acids and peroxide compounds were generated during the deep‐frying process. Furthermore, deep‐frying oil in an O/W emulsion resulted in high POV and AV values, suggesting low oxidative stability. Chemical composition of short‐term frying oil should be studied in depth in order to obtain better food quality. Considering the potential toxicity of the reaction products of thermo‐oxidative degradation of the oil, the reuse of deep‐frying oil should be considered as a public health hazard.

## CONFLICT OF INTEREST

The authors declare no conflict of interest.

## ETHICAL APPROVAL

This study does not involve any human or animal testing.
